# Supplementary figures and images for: Agent-based modelling reveals feedback loops and non-linearity between mating system evolution and disease dynamics
Source: PLoS One. 2025 Dec 18;20(12):e0336020. doi: 10.1371/journal.pone.0336020 (PMC12714262; doi:10.1371/journal.pone.0336020)

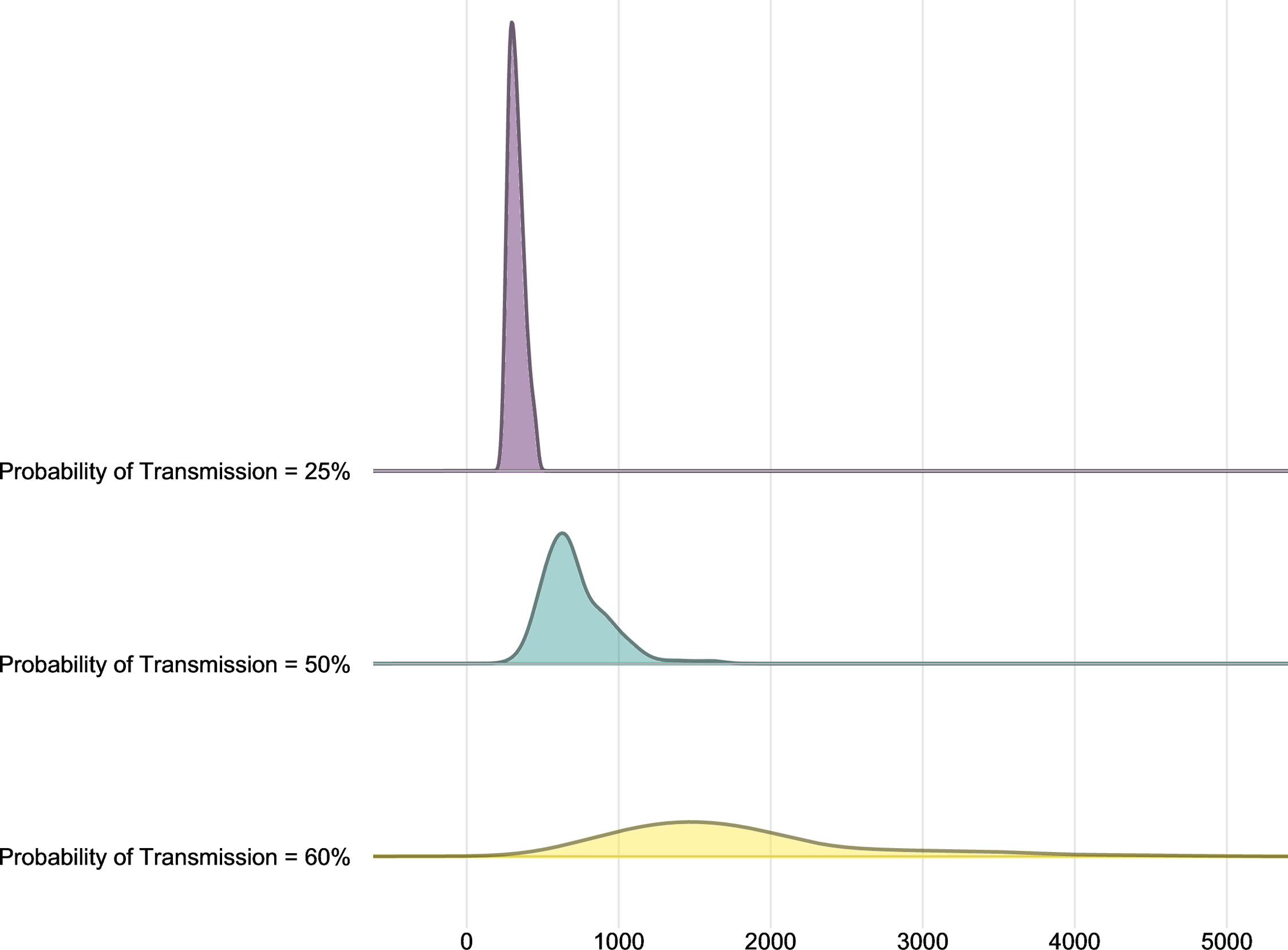

Supplement: S1 Fig — The plot reports the distributions of the time (in ticks) elapsed until the disappearance of the pathogen across all 100 runs of each setting tested in the scenario named “Minimum probability of transmission for endemization” which did not lead to endemization in a SI system (i.e., that involved probabilities of transmission < 70%). (TIF) [file pone.0336020.s002.tif]

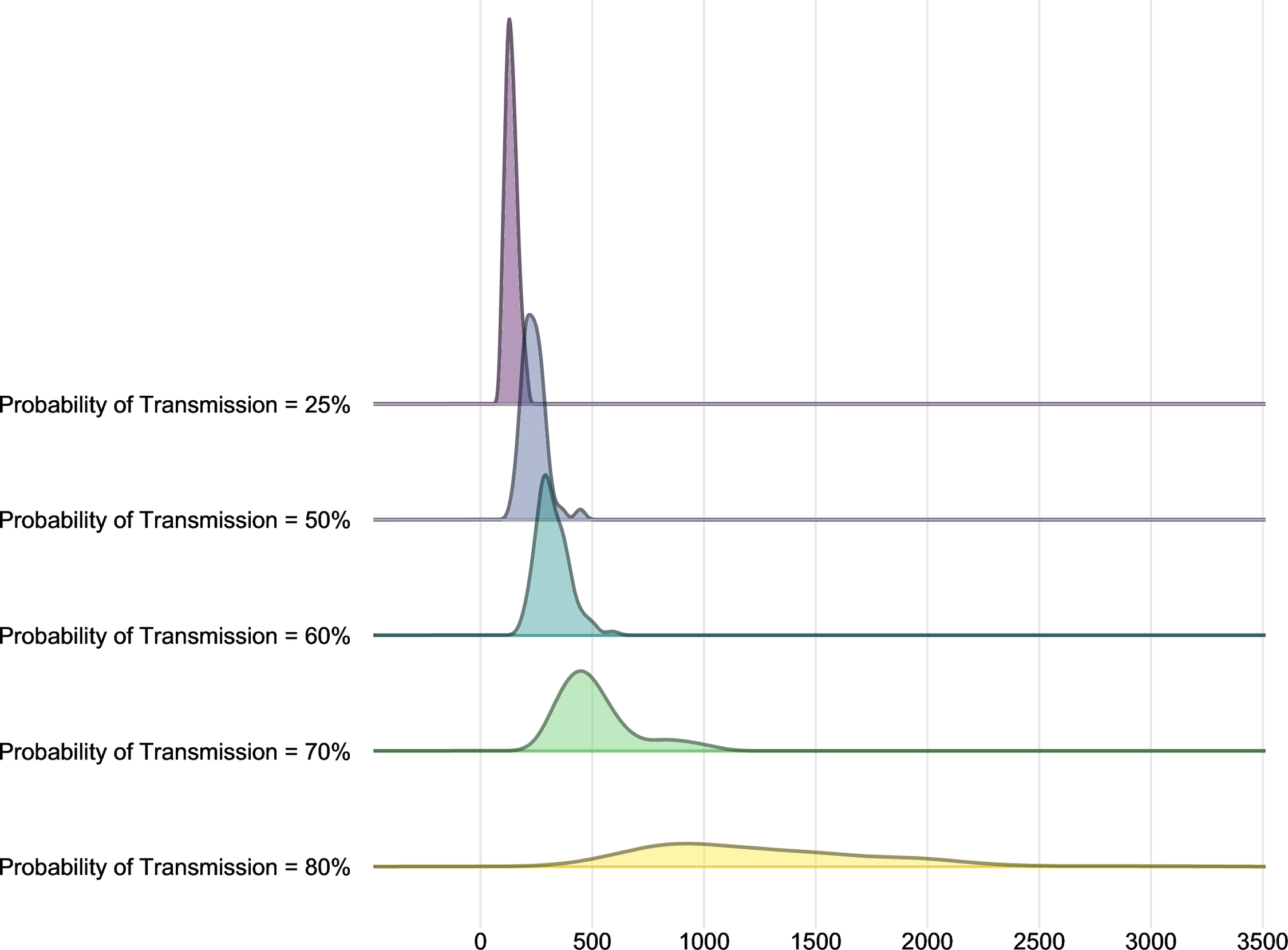

Supplement: S2 Fig — The plot reports the distributions of the time (in ticks) elapsed until the disappearance of the pathogen across all 100 runs of each setting tested in the scenario named “Minimum probability of transmission for endemization” which did not lead to endemization in a SIS system (i.e., that involved probabilities of transmission < 90%). (TIF) [file pone.0336020.s003.tif]

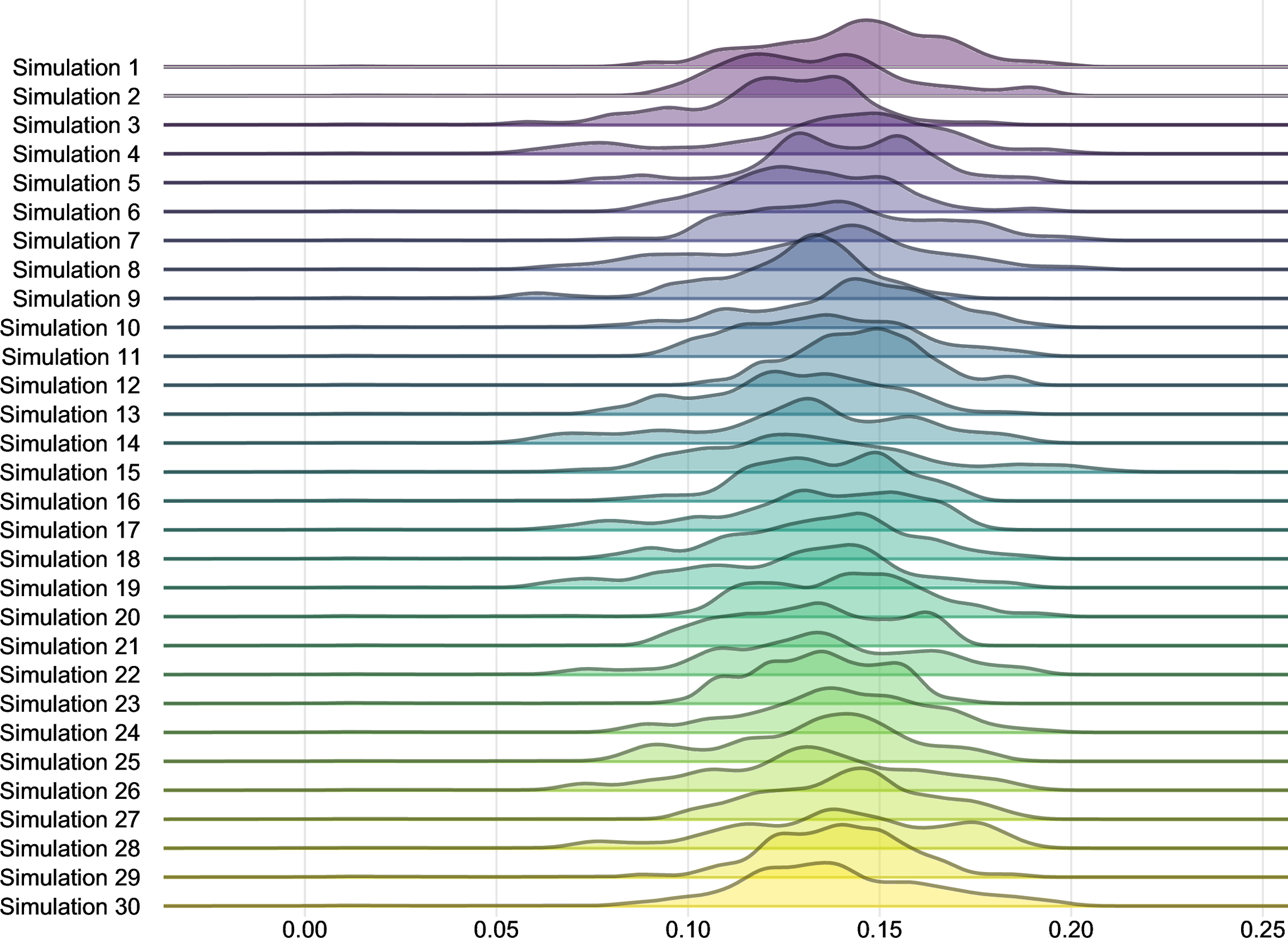

Supplement: S3 Fig — The plot reports the distributions of proportions of infected agents in the SI version of the scenario named “Minimum probability of transmission for endemization”, using the minimum probability of transmission needed to reach an endemic state in all runs (i.e., 70%). The proportions were extracted at each tick of 30 simulations, from tick 10 (the age of sexual maturity in our scenarios) up to tick 10000. (TIF) [file pone.0336020.s004.tif]
