# Supplementary material for: Agent-based modelling reveals feedback loops and non-linearity between mating system evolution and disease dynamics
Source: PLoS One. 2025 Dec 18;20(12):e0336020. doi: 10.1371/journal.pone.0336020 (PMC12714262; doi:10.1371/journal.pone.0336020)
Supplement: Attachment — Submitted filename: Response to Reviewers.docx [file pone.0336020.s006.docx]

**Response to Reviewers**

**Title of the manuscript**: Agent-based modelling reveals feedback loops and non-linearity between mating system evolution and disease dynamics

**Manuscript ID**: PONE-D-24-14535

08/02/2025

Dear Editor,

We are grateful for the opportunity to submit a revision of our manuscript entitled "Agent-based modelling reveals feedback loops and non-linearity between mating system evolution and disease dynamics", which is now revised substantially to address the comments from two reviewers.

We have revised the manuscript following all the referee's suggestions. Please find our responses on a point-by-point basis to all the comments below.

In short, we have taken on board the recommendations from both reviewers and have revamped the study by implementing the following key changes:

- We have added new simulation scenarios that contemplate recovery after infection (i.e., we run simulations not only in SI but also SIS contexts). Additionally, we also explore the consequences of vertical transmission, on top of horizontal transmission of the disease. Consequently, our models represent a more accurate representation of real-world disease dynamics in the context of sexually transmitted infections.

- We have now included new parameters/settings and extended the range of selectable values for several variables (age of sexual maturity, lifespan, time out of the market, etc.)

- We have run all simulations again, i.e., including those concerning the previous scenarios and contexts, because of the changes made to the model. In particular, we have replaced two of the original scenarios with two new ones using the new settings and functions.

- We have added an entirely new section on model applicability and limitations. We also discuss at length model validation and include additional references backing up assumptions or providing case studies.

- We have integrated into the main text the original Supporting Figures and replaced all previous figures with new ones based on data generated by the updated model.

We hope that you will agree that the manuscript has improved considerably following these revisions, and we wish to thank you, and both reviewers for taking the time to consider our work and for the very insightful comments received.

Yours sincerely,

Riccardo Tarantino (corresponding author), on behalf of all authors.

**Comment on Journal Requirements:**

As you requested, we carefully checked that our revised manuscript and material met the requirements of PLOS ONE. More specifically, we uploaded the updated version of the model (S1_Appendix.nlogo) and provided the necessary information for its use in our Data availability statement (see lines 889-893 of the revised manuscript). In addition, we checked that all figures and tables of the revised manuscript had their own captions and were cited in the text.

**Response to Reviewer 1:**

**Comment 1.0**: Dear authors, I would like to thank the authors for their submission. The study provides some new insights regarding the relationship between mating system evolution and sexually transmitted diseases. I think the study has some merits for publication, provided the authors can clarify and address the issues outlined below, particularly regarding the assumptions made in agent-based model, which are crucial for trusting the implications derived from it. Additionally, it would be helpful to discuss the major limitations of the study, allowing readers to more effectively assess the results.

**Reply 1.0**: We are grateful for the evaluation and the careful analysis of the model’s design. We have taken the suggestions on board and believe that the new version of the manuscript is much more robust and that the experimental reliability of our model is better justified. We also used some of the considerations on the limitations of our computational system to expand its functions/settings and simulate new scenarios. Indeed, we included additional options such as recovery after infection and vertical transmission of the disease from infected mothers to their offspring. For this reason, we think that the latest revisions might have significantly increased the applicability and generality of our model.

**Comment 1.1**: Could you clarify which type of epidemic model you adopted (e.g., SI, SIS)? Additionally, it would be helpful to include a discussion about the potential implications of considering other "classes of agents," such as recovery, especially given that some STDs can confer temporary immunity from infection. The choice of model structure is crucial, as it can significantly influence the dynamics and outcomes of disease epidemiology. It might also be worth mentioning this as a limitation if recovery or immunity was not incorporated into the model.

**Reply 1.1**: In the previous version of the study, we only focused on a SI model with susceptible and infected individuals, where animals contracting the infection remained infected for all their lifetime in all scenarios involving the STD. However, following the reviewers' suggestions, our updated model includes the option of running simulations within the context of a SIS system, where infected individuals recover from infection after a regulable time interval (i.e., the new variable named “*Time-to-Recover*”, see Table 1), although they do not gain immunity against the pathogen after recovery. Thus, we mentioned the absence of immunity in a new subsection of the Methods named “*Model applicability and limitations*” (see lines 359-363 of the revised manuscript) and added some new experimental scenarios/extended some previous scenarios using the SIS version of the model: “*High benefits, variable transmissibility*”, “*Variable transmissibility*, *large cost-benefit gap*”, and “*Minimum probability of transmission for endemization*” (see lines 608-632, 634-659, 546-572 in the Results and 743-775, 834-849, 777-797 in the Discussion).

**Comment 1.2**: Vertical transmission is an important component of transmission models, particularly in the context of STDs. It would be valuable to include a discussion on its potential impact on the model's outcomes and dynamics, as it could significantly influence the overall disease trajectory and population-level effects.

**Reply 1.2**: This is another comment that suggested to us a further extension of the model and some alternative/additional experimental scenarios. Vertical transmission of the infection from infected mothers to their offspring is now possible in the updated version of the model, so we used this new feature to modify and simulate again some experimental scenarios, that is, “*Variable transmissibility*” (see lines 518-543 in the Results and lines 730-740 in the Discussion, where the IA of the previous version of the manuscript is now replaced by a second SA including vertical transmission) and “*High benefits, variable transmissibility*” (see lines 608-632 in the Results and lines 743-775 in the Discussion). We also created an additional scenario named “*Variable transmissibility, large cost-benefit gap*” (see lines 634-659 in the Results and lines 834-849 in the Discussion).

**Comment 1.3**: It would be helpful to provide a more detailed description of the randomization process in your agent-based model (e.g., random number generator used (e.g., uniform, Gaussian))

**Reply 1.3**: Thank you. We agree that details on the randomization process will be relevant to the reader, so we have now added a paragraph focusing on the stochastic component of our model, including some information on the algorithm we used, i.e., the Mersenne Twister pseudo-random number generator (see lines 185-191).

**Comment 1.4**: One key assumption in your model is: “infection implies a reproductive cost for the host: everything else being equal, infected individuals generate less offspring than susceptible individuals.” While the relationship seems plausible, it would be helpful to clarify the basis for this assumption. Citing relevant references or empirical evidence to support it would strengthen the validity of the model and its underlying assumptions. Additionally, it would be beneficial to provide explanations/justifications for all assumptions made in the model.

**Reply 1.4**: In order to provide a more detailed explanation of the choice of the parameter values we used in our experimental scenarios, we added some paragraphs within the new subsection named “*Model applicability and limitations*”. In particular, the relationship between the disease and the reduced fecundity of infected individuals is now supported by several additional references and empirical evidence referred to a variety of taxa including insects, mammals, snails, and birds (see lines 335-344).

**Comment 1.5**: In the text, it was mentioned that “agents starts with age 0 and are non-sexually mature until they reach the age of 1 day/1 month/1 year (i.e., 10 ticks)”. Could you clarify the basis for selecting 10 ticks as the threshold for sexual maturity?

**Reply 1.5**: The value of 10 ticks as a threshold for sexual maturity is related to the lifespan of 150 ticks we selected for our runs. Indeed, this way we can keep a realistic ratio between adulthood and juvenile period, which could be applied to a variety of animals and life-cycles, from short-lived to long-lived species (depending on the presumed duration of each time unit in our system), as discussed in lines 322-333 of the new subsection “*Model applicability and limitations*”. For a specific example of the validity of this parameter value, see also lines 224-228 . However, in the updated version of our model, we also added a new slider that allows the user to change the age of sexual maturity (i.e., *Age-of-Sexual-Maturity*, see Table 1 and lines 220-223).

**Comment 1.6**: The upper threshold for the age of death of agents was set at 300. I am wondering how the model outcomes might change if this threshold were set to a higher value, such as 1000. Providing a justification or basis for selecting this specific threshold would be helpful.

**Reply 1.6**: As pointed out in Reply 1.5, we decided to use a fixed lifespan (= 150 ticks, i.e., 15 days/months/years) and a proportional age of sexual maturity (= 10 ticks, 1 day/month/year) in all our scenarios so that they could reflect the actual life cycle of a variety of animals. For this reason, we set at 300 the upper threshold of the parameter *Lifespan* in such a way that even the maximum selectable value does not deviate too much from the value used in our experiments. It is also important to point out that, considering the way the algorithm of the model was designed (e.g., see lines 250-254), it is likely that changing the value of this variable while keeping unchanged all the others would not significantly affect the outcomes. In fact, all females would simply complete their reproductive cycle long before disappearing from the simulation lattice, remaining non-receptive for almost all the time. On the other hand, if the lifespan is increased and one wants to maintain some proportionality between “developmental steps”, it will also be necessary to increase the time out of the market and the age of sexual maturity (which would eventually make the new settings similar to the original ones). Nevertheless, just as we made *Age-of-Sexual-Maturity* adjustable (see Table 1), we also extended the range of selectable values of *Lifespan* up to 1000 ticks and of *Time-Out-of-the-Market* up to 50 ticks, so the updated version of the model allows the exploration of a wider range of combinations between parameter values. For more details on the choice of the basic parameter values we used in our scenarios, see also lines 322-333.

**Comment 1.7**: How did you validate your model? I believe this is a crucial aspect of the modeling process, as it ensures we can trust the implications the model provides. It may have been mentioned in the text but could benefit from further emphasis.

**Reply 1.7**: We agree that model validation is a critical part of agent-based modelling, since the reliability of a model depends on its capacity to reproduce some relevant aspects of reality that are related to our questions. Of course, this depends both on the correspondence between the behaviour of agents and that of their real-world counterparts (*microvalidation*) and on the correspondence between patterns generated by simulations and real patterns and aggregate properties which have been described, for instance, in empirical studies (*macrovalidation*) (Wilensky and Rand 2015) (see references at the bottom of the document). In our manuscript, we provided several justifications and references for the behaviour of our agents and the choice of parameters/parameter values (e.g., see lines 150-152, 155-157, 211-231, 254-256, or 283-284), including those mentioned in the new subsection on model applicability and limitations (see lines 322-344). Furthermore, some basic experimental scenarios were specifically designed to generate data which, at least from a qualitative point of view, could be easily compared with some widely studied real-world phenomena and patterns (i.e., “*Baseline condition*”, “*Variable costs*”, and “*Who carries the pathogen*”, see Table 2A and 2B). However, we also agree that it could be important to put more emphasis on this aspect, so we added two paragraphs in the Discussion (see lines 702-709 and lines 723-728) where we explicitly mention the function of these scenarios from a model validation perspective and add some further references which strengthen the reliability of our outcomes. More specifically, these scenarios were aimed at showing the effects of costs of mating in reducing the spread of polyandry (and enhancing monandry), and the strong association between the polyandrous strategy and STDs.

**Comment 1.8**: I am not sure I fully understood what you were trying to express in Equation (2). Should it be -N_i⋅i⋅o instead of -(N_i+i)⋅o?

**Reply 1.8**: The current formula accurately reflects the calculation of the offspring number in our simulations. Indeed, it is important to point out that a susceptible female can still suffer offspring reduction if mating with infected males, even if she remains uninfected due to a probability of transmission per sexual contact different from 100%. For further details on this point, see also the new lines we added to the manuscript to clarify this mechanism (lines 293-295).

**Comment 1.9. Minor comments:**

1. Line 143: Refer to the section discussing the scenarios

2. Line 219: think Equation (1) can be simplified as: N_0=100+N_m Δ_m, where Δ_m denotes the % difference in the benefits and costs of mating

3. In Tables 2A–2C, you might consider removing the 'Experimental Scenario' row and using the text in that row as the heading for the first column instead.

4. Line 303: Better to use the word replication instead of replica.

**Reply 1.9 (minor comments)**: We edited the manuscript following all the indications. Thank you very much.

**Response to Reviewer 2:**

**Comment 2.1**: Not including a recovery state in the simulation is problematic since animals can recover from many STDs. For example, some animals can develop resistance to infections over time, and recovery can significantly impact disease dynamics. Including recovery rates would provide a more accurate representation of real-world scenarios and enhance the model's applicability. This should be included in the model and additional simulations should be made.

**Reply 2.1**: We agree that including the ability to recover from infection could be a relevant addition to our study, whose results were previously informative only about systems where infected individuals remain infected for all their lifetime (i.e., Susceptible-Infected or SI systems). Although SI models can describe the dynamics of a good number of STDs (see lines 308-320 and Fig 1), we also believe that the generality of our findings could be increased by adding the ability to recover from infection. For this reason, in the updated version of the model, we added the option of running simulations in the context of a SIS (Susceptible-Infected-Susceptible) system (see lines 171-175), which includes the recovery of infected individuals after a time interval that is regulated using the new slider named “*Time-to-Recover*” (see Table 1 and lines 301-303). We also used this new function of the model to design and simulate some alternative/additional scenarios, that is, “*High benefits, variable transmissibility*”, “*Variable transmissibility*, *large cost-benefit gap*”, and “*Minimum probability of transmission for endemization*” (see lines 608-632, 634-659, 546-572 in the Results and 743-775, 834-849, 777-797 in the Discussion). Most importantly, we compared the outcomes of the previous SI scenario named “*Minimum probability of transmission for endemization*” with those of the corresponding SIS scenario and found that both systems were characterized by a threshold of the probability of transmission above which the circulation of the pathogen remained constant over time in all or nearly all runs. We believe this emerging pattern could support a broader applicability of our key results to a variety of diseases and taxa. However, since we have not included immunity after recovery from the infection, we mentioned this feature as a limitation of our model in a new subsection of the Methods named *Model applicability and limitations* (see lines 359-363).

**Comment 2.2**: The reproductive benefit and cost percentages are good additions to the model. However, assuming fix values for these parameters is too simplistic. Additional simulations should be made using assumptions that are more applicable for these parameters. For example:

a) Trade-offs in Drosophila: Enhanced reproductive efforts in Drosophila result in a shorter lifespan, as the energy expended on courtship, egg laying, and mating takes a toll.

b) Parental Investment in Birds: Research on birds indicates that greater parental investment in current offspring can diminish the parents' future reproductive success and survival rates.

**Reply 2.2**: Although we recognize that this comment suggests some highly relevant applications and case studies, we believe that such a goal would be out of scope in the context of our research. Indeed, the main intention of our study was exactly that of providing a general model which, precisely because of its simplicity, may be effective in detecting non-linear relationships and counterintuitive dynamics which might be inherent to many animal systems based on the reciprocal interplay between competing mating strategies and a sexually transmitted disease. In other words, we think that the fascinating points mentioned in this comment (of which we are aware because, for instance some of our previous works relate to life history reproduction-longevity/survival trade-offs in Drosophila and other animals), would deserve to be addressed, for instance, in a study aimed at investigating the effects of temporal variations in the costs and benefits of mating, also including costs and benefits other than reductions/increases in fecundity. Keeping in mind the topics suggested by this comment, we added a paragraph where we highlight the relevance of models focusing on fertility or fecundity costs induced by STDs (see lines 335-344) and a paragraph where we point out the numerous factors which may modulate the dynamics of this kind of systems (see lines 346-357). Both paragraphs are included in the new subsection dedicated to the applicability and limitations of our model. That said, following the reviewer's suggestion to move beyond simulating simplistic fixed cost/benefit values, we now investigate some additional combinations of costs and benefits of mating. More specifically, we changed the value of the cost of mating in the scenario named “*High benefits, variable transmissibility*” (see Table 2A, lines 608-632, and lines 743-775) and added a new scenario named “*Variable transmissibility, large cost-benefit gap*” to explore an experimental condition characterized by a more pronounced gap between costs and benefits of mating (see Table 2B, lines 634-659, and lines 834-849).

**Comment 2.3**: Line 171: "we consider a susceptible-infected system, without recovery for infected."

Comment: The model does not account for recovery from infection, which is a significant factor in the dynamics of many sexually transmitted diseases. Including recovery rates could provide a more accurate representation of disease dynamics.

**Reply 2.3**: Thank you. Yes, we completely agree. In the updated model, simulations can be run either in the SI or the SIS version. The latter option enables the use of a new slider named *Time-to-Recover*, that is used to select the time interval, in ticks, from when an agent contracts the infection to when it recovers from the infection and becomes susceptible again (see Table 1). Consequently, as mentioned in Reply 2.1, we added to our study some alternative/new experimental scenarios using the SIS version of the model, namely “*High benefits, variable transmissibility*”, “*Variable transmissibility*, *large cost-benefit gap*”, and “*Minimum probability of transmission for endemization*” (see lines 608-632, 634-659, 546-572 in the Results and 743-775, 834-849, 777-797 in the Discussion).

**Comment 2.4**: Line 175: "Infection implies a reproductive cost for the host: everything else being equal, infected individuals generate less offspring than susceptible individuals."

Comment: The model assumes that the benefits and costs of mating are fixed percentages. In reality, these values can vary widely depending on environmental conditions, individual health, and other factors. This simplification might limit the applicability of the findings to real-world scenarios and even for hypothetical scenarios outlined by the model.

**Reply 2.4**: As partially discussed in Reply 2.2, our main aim with this research was that developing a computational system which could provide some relevant, general insights applicable to a variety of case studies. Of course, using fixed values of benefits and costs of mating within each experimental scenario, that is, excluding possible variation induced by changes in environmental conditions, individual health, and other variables, is indeed a limitation (as discussed in the subsection on the applicability and limitations of the model, see lines 346-357). Nevertheless, we are convinced that the presence of such limitations could also bring some advantages when dealing with agent-based models aimed at investigating general trends and patterns like the ones we included in our study. As a matter of fact, the general issue from which these considerations arise is related to the philosophy underlying computational modelling. In order to be usable and capable of generating some insights, a model must necessarily isolate some key variables and make relevant simplifications of reality. Thus, every model is inevitably “false”, at least to some extent (this concept is perfectly exemplified by George E. P. Box’s famous motto “All models are wrong, but some are useful” (Box 1976). The inverse relationship between the level of realism and the intelligibility of a model representing a complex system is also well described by the various formulations of Bonini’s paradox (e.g., see (Dutton and Starbuck 1971)). In other words, we feel that some of the insights suggested by the current version of our agent-based model would be overlooked by using a highly complex model focusing on variables like those suggested in this comment, which obviously does not rule out the relevance of a future model focusing on other factors like those mentioned above.

**Comments 2.5**: Line 146: "The key features of the model are applicable to a wide range of taxa with sexual reproduction, including many invertebrate and vertebrate species."

Comment: While the model is designed to be applicable to a wide range of taxa, the specific parameters and behaviors chosen may not accurately reflect the biology of all species. For example, the assumption that all males are polygynous might not hold true for species with different mating systems.

**Reply 2.5**: Of course, the parameter values we used in our experimental scenarios would not accurately represent all animal species showing multiple mating strategies associated with females, because the existence of (even many) exceptions is clearly another intrinsic characteristic/limitation of theoretical modelling. Nevertheless, we are also convinced that the level of generalization of our model and scenarios (see also Reply 2.4) makes them largely capable of providing some useful information about a wide variety of animal systems. We discuss in detail this aspect in lines 308-344 of the new subsection on model applicability and limitations. As for the specific example of males’ polygyny, as we pointed out in the Methods section, and bearing in mind that we used the term "polygyny" as male multiple mating (Gowaty 2012), this assumption would be valid for a very large number of species, as evidenced by a large body of literature on sexual selection and mating systems (see lines 155-157, and for instance (Andersson 1994; Birkhead and Møller 1998; Simmons 2001; Shuster and Wade 2003)).

**Comment 2.6**: Line 169: "For the same reason, we did not create a further class of agents for the pathogen."

Comment: The model's reliance on a hypothetical pathogen means that the results may not be directly applicable to any specific real-world disease. The authors only considered an SI system without recovery which is not applicable to many sexually transmitted diseases.

**Reply 2.6**: The most relevant characteristic of the hypothetical pathogen considered in our study is its probability of transmission per sexual contact, that is a typical variable associated with sexually transmitted diseases, regardless of the specific pathogen involved. Adding characteristics which are applicable to a particular pathogen would not have been informative in the context of a general model investigating the interplay between mating systems and epidemic dynamics. Even worse, it could have made the model only applicable to one or a few case studies, greatly limiting the generality of the patterns detected. In other words, our aim was not to develop a model for a particular pathogen. Rather, we were interested in epidemic dynamics at a population level that are largely independent from the peculiarities of a specific pathogen underlying the disease. For more details on the applicability of the SI version and the new SIS version of our model, see Reply 2.1, 2.3, and lines 308-320 in the revised manuscript, including Fig 1.

**Comment 2.7**: Line 278: "We conducted Symmetric Analyses simulations, henceforth 'SA', where the initial number of M and P females is the same, that is, 50 agents for each strategy."

Comment: The impact of varying initial conditions, such as different starting ratios of males and females, and monandrous to polyandrous females, could be explored in more detail to understand how initial population structure influences the results.

**Reply 2.7**: This is an interesting question we also asked ourselves while defining the experimental scenarios to be simulated and included in our study, in particular, focusing on the initial sex ratios. However, preliminary analyses performed with the model suggested that changing the initial sex ratio had no relevant impact on the evolution of the system, most likely because, even from the F_1_ generation, the population undergoes a marked rebalancing of sex ratio (see also lines 407-410). This is the main reason why we did not include such scenarios and decided to focus on other more impactful variables. As for the monandrous-polyandrous ratio, we were mainly interested in investigating the effects of very marked imbalances between the two strategies through our Invasibility Analyses (IA), where we simulated the invasion by a single M strategist (e.g., see lines 470-473).

**Comment 2.8**: Line 503: "Our agent-based modelling approach to the study of the interrelationship between mating strategies and epidemiological dynamics informs that this interplay may be remarkably complex and may have important repercussions for both the spread of disease and the evolution of sexual behaviours and mating systems."

Comment: The implications of the findings for managing sexually transmitted diseases in wildlife or human populations are not fully discussed. Addressing the ethical and practical considerations of applying these insights in real-world contexts would be valuable. Additionally, implying that a simple agent-based model has important repercussions must be backed up with model data validation from real-world data. This would enhance the credibility and applicability of the findings.

**Reply 2.8**: We refer to Reply 1.7 on model validation. In short, we agree that model validation is critical and are sorry that the previous version of the manuscript was unclear with this important aspect. In the revised manuscript we provide several justifications and references for the behaviour of our agents and the choice of parameters/parameter values (e.g., see lines 150-152, 155-157, 211-231, 254-256, or 283-284), including those mentioned in the new subsection on model applicability and limitations (see lines 322-344). In addition, some basic experimental scenarios were specifically designed to generate data which, at least from a qualitative point of view, could be easily compared with some widely studied real-world phenomena and patterns (i.e., “*Baseline condition*”, “*Variable costs*”, and “*Who carries the pathogen*”, see Table 2A and 2B). We have also added two paragraphs in the Discussion (see lines 702-709 and lines 723-728) where we explicitly mention the function of these scenarios from a model validation perspective and add some further references which strengthen the reliability of our outcomes. Finally, we add a small paragraph with some considerations on the general practical applications of our findings (see lines 869-878). We hope the reviewer agrees that these are satisfactory changes. We again thank the referee for very insightful comments and useful recommendations.

**References cited in this document**

Andersson, M. 1994. *Sexual selection*. Princeton University Press, Princeton.

Birkhead, T. R. and A. P. Møller, eds. 1998. *Sperm competition and sexual selection*. Academic Press, San Diego, California.

Box, G. E. P. 1976. Science and Statistics. *Journal of the American Statistical Association* 71:791-799.

Dutton, J. M. and W. H. Starbuck. 1971. *Computer Simulation of Human Behavior*. Wiley.

Gowaty, P. A. 2012. The evolution of multiple mating: Costs and benefits of polyandry to females and of polygyny to males. *Fly* 6:3-11.

Wilensky, U. and Rand, W. 2015. *An Introduction to Agent-Based Modeling: Modeling Natural, Social, and Engineered Complex Systems with NetLogo*. The MIT Press, Cambridge, Massachusetts.

Shuster, S. M. and M. J. Wade. 2003. *Mating Systems and Strategies*. Princeton University Press, Princeton, New Jersey.

Simmons, L. W. 2001. *Sperm Competition and its Evolutionary Consequences in the Insects*. Princeton University Press, Princeton.
