# Supplementary material for: Agent-based modelling reveals feedback loops and non-linearity between mating system evolution and disease dynamics
Source: PLoS One. 2025 Dec 18;20(12):e0336020. doi: 10.1371/journal.pone.0336020 (PMC12714262; doi:10.1371/journal.pone.0336020)
Supplement: Attachment — Submitted filename: Response_to_Reviewers_auresp_2.docx [file pone.0336020.s007.docx]

**Response to Reviewers**

**Title of the manuscript**: Agent-based modelling reveals feedback loops and non-linearity between mating system evolution and disease dynamics

**Manuscript ID**: PONE-D-24-14535R1

20/09/2025

Dear Editor,

We are grateful for the opportunity to submit a new revision of our manuscript entitled "Agent-based modelling reveals feedback loops and non-linearity between mating system evolution and disease dynamics".

Reviewer 1 stated that his/her previous comments were addressed sufficiently in the previous submission. Thus, all changes introduced in this updated version of the manuscript have been included with the aim of addressing the recommendations and suggestions we received from the Editor and the third reviewer. Such comments have allowed us to refine several aspects of our work, and at the same time helped us to present our results more appropriately and clearly. Please find our responses on a point-by-point basis to all the comments below.

In a nutshell, we have improved our study by implementing the following key changes:

- We have replaced some of the older references with more recent ones, including reviews where available, to provide a more balanced and up-to-date representation of the literature.
- We have added a new feature to our model, that is, a counter that reports the number of sexual contacts of polyandrous females during simulations, and expanded on some of our findings by performing some additional experiments. We have also replaced/added paragraphs in the manuscript to contextualise or clarify some of our previous statements or arguments.
- We have run most of our experimental scenarios again, this time performing 500 simulations instead of 100, to make our outcomes more reliable. We have also significantly improved some plots, e.g., using paired boxplots instead of bars to compare the times to fixation of the two competing genotypes, and added new figures aimed at showing the distributions of the data generated by several scenarios. More specifically, we have replaced Figures 2, 3, 4, 5, 9, and 10, and added S1, S2, and S3 Figures.
- We have merged the Results section and the Discussion section into the new section entitled “Results and Discussion” to make our findings easier to understand, as suggested by the reviewer.

As noted, detailed explanations of how we have individually addressed each of the reviewer’s comments are provided below.

We hope that you will agree that the manuscript has been further strengthened thanks to these revisions, especially in terms of the communication of the outcomes generated by our simulations.

Once again, we wish to thank you and all reviewers for taking the time to consider our work and for all the constructive, insightful comments we have received during the process.

Yours sincerely,

Riccardo Tarantino (corresponding author), on behalf of all authors.

**Responses to Reviewer #3:**

**Comment 0**: This manuscript details a simulation of the relationship between female mating strategies and female-sterilising STDs. The manuscript then explores the how varying some aspects of the models parametrisation effects the success of a multi-mate versus single-mate strategy, and how this impacts the persistence of STDs and compares the two. These findings are interesting and publishable but there are issues with how these are communicated, I have divided my comments between major and minor issues that undermine the rigour of this analysis, and other comments that relate to the content but shouldn’t undermine this manuscript being published in PLOS One.

**Reply 0**: We wish to thank the reviewer for the evaluation and the careful analysis of the results of our simulations. We also agree that it is highly relevant that all findings contained in the manuscript are presented as precisely and clearly as possible, so we welcome with sincere interest all the observations listed in these very detailed comments. In the new version of the manuscript, we have tried to implement as many suggestions as possible, and we believe that the resulting changes could constitute a significant improvement that will make clearer the link between the outcomes of our simulations and the conclusions we have proposed in the text. In particular, we have updated the model and run a large number of additional simulations, both to support our conclusions on the original scenarios and to clarify or investigate further several aspects of our system which were previously unexplored. This has also led us to improve the quality of many of our plots and create some new figures to show the distributions of our data in several scenarios. Furthermore, we have reorganised the text to make it more readable, clarified the ambiguous passages by expanding our reasoning or refining our claims, and updated some bibliographical references. We hope that the reviewer will appreciate the modifications we have made.

**Major comments**

**Comment 1**: A (acknowledged) limitation of this model is the lack of immunity or a “recovered” compartment in the SI and SIS structure. This is a fair assumption for many STIs but not everyone, for some reason the authors included fig 1 which attempts to list which STDs for which an SI or SIS model would be useful. It includes very questionable choices, such as HPV for SI and SIS and campylobacter, a confusing mix of vector-borne diseases that are STDs in vectors (and also in hosts) and is cited in a way that makes it very difficult to follow-up citations. Why include this table? It is completely unnecessary, the authors could have just provided examples to justify the use of SI and SIS model structures.

**Reply 1**: We acknowledge that the ascription of certain STDs to a specific epidemiological model may be debatable, but all the examples we present are documented in the literature (we cited some key reviews that cover most examples). Moreover, the fact that some of these cases involve vector-borne diseases that are sexually transmitted in the vectors is not a drawback; rather, it illustrates the broad incidence and diversity of contexts in which STDs can occur. We would therefore prefer to retain Figure 1, as it demonstrates that the outcomes generated by our model can be applied, in general terms, to a broad spectrum of cases. Indeed, the figure was originally included precisely to emphasise this generality, in response to comments from reviewers in the previous round concerning the generality and applicability of our model.

**Comment 2**: The authors discuss STDs generally in the introduction and the title but the models exclusively focuses on STDs that might sterilise female infected, the title and introduction should be updated to reflect this. The discussion however does make this clear. In table 1 and the methods section so attempt is made to justify this choice (which is fair, many STDs are sterilising) however the citations chosen often refer to diseases which are diseases which also have detrimental impacts of the livelihood of infected animal and it raises why these aspects are ignored in the model. The authors should clarify their choices to focus on STDs that only impact fertility and correct their citations. Also some citations refer to the evolutionary cost of immune-system which is different to the costs of an infection, and not relevant to what the authors model captures

**Reply 2**: Thanks for the comment. With all due respect, we would like to emphasise that our focus is on the costs of infection in terms of reproductive success. From an evolutionary perspective, what matters most are the gains and losses in reproductive success, and the overall balance. Our model incorporates this ultimate measure by concentrating the detrimental effects of infection on reproductive costs that are expressed as a reduction in offspring production (i.e., a decrease in reproductive success). This point is conveyed in several parts of the manuscript (e.g., lines 184–186, 289–295, and equation 1), but we have now clarified it further (lines 186-190). Moreover, our aim was to develop a general model whose outcomes could apply across a wide variety of taxa, conditions, and scenarios. Taking this summarising perspective allows broader extrapolation. More complex or specific pathological conditions—for example, cases where infection simultaneously affects fertility and longevity—are certainly interesting, but they apply to a narrower spectrum of cases.

As for the second problem identified by the reviewer concerning the costs of the immune system, we agree that this is a problem and are grateful to the reviewer for pointing this out. We have now removed the citations that could be misleading. We have, however, retained the comment "*Furthermore, physiological costs associated with immune defence are expected, and infections may reduce reproductive output through trade-offs between resource allocation to immunity defence and reproductive effort*", given that it illustrates our point above: it is not the cost of infection what we refer to here, but the ultimate consequence of infection for reproductive output (either from direct effects of infection, or through the expression of trade-offs that are exposed by infection).

**Comment 3**: There is a comical trend amongst computational evolutionary biologists working on infectious diseases to rediscover R0 every time they publish a paper. R0 is well defined in endemic diseases (though often interpreted through Rt or Reff in relation to immunity). Just because your model is much simpler, this doesn’t make this the discovery of a new threshold. If you want to explore how this threshold relates to sexual strategy you could actually explore that by recording the number of sexual contacts in your model, it would be quite interesting.

**Reply 3**: We thank the reviewer for this important hint and agree that the endemic threshold presented in our manuscript could actually represent a situation where R_0_ = 1 and the spread of the pathogen tends to be stable over time. Consequently, we also recognise that some of our claims needed to be scaled back and contextualised, so we have modified lines 40-42 in the Abstract and added lines 682-685 in the new *Results and Discussion* section. We believe that the outcomes generated by this scenario are still relevant because of the particular (although still broadly applicable) conditions we used, since we considered simultaneously: i) a sexually transmitted disease with variable rate of transmission, ii) a fully polyandrous context, iii) panmictic mating, iv) male polygyny, v) a randomly selected degree of polyandry per female, and vi) a refractory period to further copulations which is applied to each female after mating. In addition to these settings, we also focused on comparing the behaviour of the system between the two alternative versions of the agent-based model, SI and SIS. Therefore, the new/modified lines of the manuscript referring to this scenario (especially lines 666-685) emphasise the specificity of the results shown.

We also greatly appreciate the reviewer’s suggestion to explore this scenario further and have supplemented our previous results on endemic thresholds with an exploration of the total number of sexual contacts observed during each run. To allow this additional investigation, we have modified the model by introducing a new variable named *Sexual-Contacts*, whose values are reported in the NetLogo interface by a monitor called “*Sexual Contacts (P Females)*”. This monitor is specifically used to keep track of the number of matings between polyandrous (P) females and males during simulations (see lines 313-316). We have not included the number of matings of monandrous (M) females because they are not involved in the scenario we wanted to expand. We believe that this new variable and the simulations in which we have monitored it could enrich the investigation focusing on the attainment of endemic states (given an ecological context that exhibits the characteristics listed above) with further useful and surprising details, which are presented in lines 687-694 and 704-719.

In a nutshell, this new SI scenario uses the same settings as the “*Minimum probability of transmission for endemization*” scenario (see Table 2C), except for the use of a couple of alternative values of *Time-Out-of-the-Market* (2 and 18 ticks) in addition to the default value that we have always used in this study (6 ticks), and of a single value of *Probability-of-Transmission-%*, i.e., 70%, which is the endemic threshold in our SI model. As suggested by the reviewer, we have monitored the total number of sexual contacts associated with the polyandrous genotype/strategy in a context where we previously observed a stable persistence of the pathogen over time in all runs.

Interestingly, we have found that neither reducing the refractory period of P females by two-thirds nor tripling it prevented the reaching of a stable pathogen circulation over time, which still occurred in all runs. Surprisingly, a relevant reason why a 70% probability of transmission seems to be sufficient to guarantee endemization across the three different conditions explored in this additional test may lie in the fact that the total number of matings recorded at the end of every run, i.e., after 10000 ticks (1000 days/months/years) was remarkably stable around 269000. More specifically, using a refractory period of 2 ticks, the mean number of sexual contacts across runs was 269553.51 (SD = 756), while this average value was 269137.91 (SD = 602.39) and 269673.29 (SD = 651.07) when using refractory periods of 6 and 18 ticks, respectively. Thus, changing the rate of production of new batches of offspring by modifying *Time-Out-of-the-Market* (in the model, oviposition by P females always occurs at the same time as mating) might neither reduce nor increase the number of contacts significantly, which could make the duration of P females’ refractoriness to new matings irrelevant in this specific context, implying that the endemization threshold detected here is potentially shared by many animal systems with refractory periods of different magnitude. We believe that this is an important extension of our original result, which helps reading the previous outcomes in a new light.

**Comment 4**: I don’t think that the conclusions on 825-829 are supported by your simulations. All the lower chance of success with a lower initial population indicates is that the smaller populations are more susceptible to stochastic effects. I’m not sure how the following line proceeds from that, please expand on your reasoning.

**Reply 4**: We concur with the reviewer that the reason why P strategists tend to become extinct in most runs when they start at a numerical disadvantage is the positive relation between their initial frequency and their probability of fixation, as they are of course more subject to stochastic effects and closer to the reaching of that absorbing state. We have also tested whether this effect was specifically due to the small initial population size of P or, as we have just mentioned, to the initial numerical inferiority of P compared to M. To do this, we have performed a further alternative scenario with 1900 M and 100 P and found that the outcomes are similar to those obtained with 190 M and 10 P (which are reported in lines 766-771). The relative frequency of fixation of P in these two scenarios using 100 runs was 11% and 5%, respectively, which suggests that the initial numerical inferiority of P induced by the epidemic, regardless of population size, is a plausible cause of the pattern that we have reported.

Indeed, our main aim in the lines describing the additional simulations related to the experimental scenario under examination (see lines 760-774, especially from line 771 onwards) was to point out that a simple mechanism based on the frequency of genotype P in the absence of an epidemic was a relevant component in explaining an apparently counterintuitive pattern, taking into account the strong association we previously reported between the P strategy and STDs. For greater clarity, we specify that the counterintuitive pattern referred to here and in the manuscript is the high frequency and temporal proximity with which the polyandrous genotype becomes extinct after the pathogen has disappeared from the population (for further details, see lines 723-736 in Results and Discussion), although P females are the main victims and carriers of it.

In this regard, we must recognise that the passage commented on by the reviewer may appear controversial and seems to place excessive emphasis on a process that we did not intend to present as unprecedented, but just as one of the plausible mechanisms affecting this unexpected behaviour of the system. For this reason, we felt it was important to amend the text and provide a more accurate, extended explanation of our claims (see lines 774-790). We hope that the new, more detailed and comprehensive argument will significantly dispel the ambiguity which was correctly pointed out by the reviewer.

As for the connection between this paragraph and the beginning of the next one in the previous version of the manuscript (original line 831), we must point out that these two paragraphs are no longer contiguous due to the reorganisation of the text that we have carried out following the reviewer’s suggestion (see Comment and Reply 16). However, it is important to note that our intention when we talked about “*another* unexpected behaviour” (original line 831) was exclusively to emphasise that both results mentioned presented some counterintuitive elements, although the scenario used to produce the second of these results included settings very different from those used in the scenario on which the reviewer’s comment focuses.

**Minor comments**

**Comment 5**: Some potential sensitivities are ignore and should be discussed or explored, in particular the impact of population threshold on model findings.

**Reply 5**: The decision to use specific values for certain variables across all scenarios was motivated by preliminary checks of the model’s functioning. Indeed, we noticed that the most relevant parameters affecting the collective behaviour of the system were those that we manipulated in our simulations, i.e., *Probability-of-Transmission-%*, *Benefit-of-Mating-%*, *Cost-of-Mating-%*, and *Offspring-Reduction-%* (induced by the effects of the pathogen). Exploring the effects produced by gradual changes in the values of these few key variables led to all the results presented in our manuscript.

In contrast, the values of other variables used to define basic characteristics of the agents, such as *Lifespan* and *Age-of-Sexual-Maturity*, were used to reflect (in proportion) the life cycle of a hypothetical animal species, as we mentioned in lines 217-237. An exhaustive analysis of the sensitivity of most of those parameters would not be particularly useful if our goal is to represent a wide variety of plausible contexts. As for *Time-Out-of-the-Market*, we decided to “*keep it fixed at 6 ticks for all our simulations because we found that this interval is appropriate to ensure longer coexistence and equal probabilities of fixation for each mating strategy, everything else being equal*” (lines 274-277), and variation around this value does not seem to have any relevant effect on the epidemic dynamics, at least as far as the attainment of endemic states is concerned (see Reply 3 above).

However, we agree that the effect of variation in the *Juvenile-Threshold* requires attention. To this end, we have now run new simulations of the scenario “*Minimum probability of transmission for endemization*” using four different values of *Juvenile-Threshold* (500, 1000, 1500, and 2000 coexisting juvenile individuals) and not only one value (1500), as in our previous version of the study. Results show that while upper values of the maximum number of juveniles for each tick lead to endemic states in 100% of the runs (a result that confirms our previous findings), a value of 500 only leads to endemization of the STD in 74% of the runs. So, we can conclude that indeed small populations make a difference (see also Reply 10). We present in the new version all these points (see lines 431-433 and 687-702).

**Comment 6**: Simulation results are poorly plotted. The authors should consider including binomial proportion confidence intervals in their plots to account for the small simulation size used. The bar plots don’t need to depict both counts summing to 100, but should just show the proportion where M (or P) is dominant along with the confidence intervals. In general I find some of the comparisons unconvincing due to the small number of simulations so it would help support comparisons to have some measure of certainty given the simulation counts (or you could run more simulations). In addition the time to fixation plots within the same figure should be plotted on the same axis, i.e. fig2 B and D should both be plotted on a scale of 0-550 days/months/years. It would also be good to get an idea of the distributions around time to fixation using violin or boxplots rather than bar plots of the mean.

**Reply 6**: We are grateful for these important suggestions and have tried to implement most of them to improve the quality of many of our plots as much as possible, in order to achieve a better communication of the results of our scenarios. More specifically, we have run many more simulations for most of our scenarios (i.e., 500 runs for each specific setting of each scenario, instead of the previous 100), adjusted the axes, and replaced bar plots with boxplots to give an idea of the distributions around times to fixation. The graphical representations of the relative frequency of fixation, although based on a much higher number of simulations, have remained largely unchanged. We believe that depicting this relative frequency using bar charts is the most immediate way to display our outcomes from a visual point of view. The updated figures are Fig 2, Fig 5, Fig 9, and Fig 10. Of course, the parts of the manuscript where we mention the outcomes of these updated experiments have also been updated/corrected accordingly (see subsections *System’s response to variations in costs of mating*, *Epidemic dynamics* from line 576 onwards, *Medium-low transmissibility reveals a switch-like behaviour when polyandry is highly beneficial*, and *Effects of variable transmissibility when the cost-benefit gap is large* in Results and Discussion).

**Comment 7**: I don’t understand the point of the equation f_m = 1/N on line 470?

**Reply 7**: That is right, that equation is unnecessary. We have removed it.

**Comment 8**: In figure 3 you compare the correlation of infections against the population of P females. Wouldn’t be to better to just compare the proportion of P females that were infected against the proportion of M females that were infected. I don’t understand why you’re using an abstraction here? You might need to adjust for the proportion of time at risk if there are STD/strategy extinctions but then you could just calculate the STD prevalence by female time(ticks) at risk. The same holds for Figure 4, why not plot the number of p-females infected and the number of m-females infected instead of comparing trends. You have a complete simulation you don’t need to use the limited approaches we have for observational data.

**Reply 8**: We agree with the reviewer that this alternative approach could allow readers to grasp the close relationship between the polyandrous strategy and the disease more clearly and directly, so we have replaced the original Fig 3 and Fig 4 with a ridgeline plot representing the distributions of proportions of infected P and M females in 30 simulations and a line chart representing the number of infected P and M females during a single simulation, respectively (see lines 525-559 for the new results of the scenario “*Who carries the pathogen*”).

**Comment 9**: How many simulations where used for Figure 6 and can we have some indication of the distribution of this?

**Reply 9**: When the selected value of *Probability-of-Transmission-%* was tested using both model structures, that is, when *Probability-of-Transmission-%* = 25%, 50%, 60%, and 70%, we executed 200 simulations (100 simulations for the SI model and 100 simulations for the SIS model). In contrast, we executed 100 simulations for the two values tested using only the SIS model (i.e., 80% and 90%). We have now specified the number of simulations used for each scenario in the updated captions of Table 2 (A, B, and C). The distributions of our data are now shown in S1 Figure and S2 Figure (mentioned in lines 635-638).

**Comment 10**: Figure 7, would be good to show the distributions of this from many simulations (plus a single line so we can have some idea of the trends within a single simulation). As an aside such a low level of prevalence for a life long disease suggests the number of mating events occur relatively slowly compared to the replenishment of susceptible, it would be interesting to see the sensitivity against the number of new born, I’d guess the results will change quite significantly.

**Reply 10**: We have created the new S3 Figure (mentioned in lines 652-656) to show the distributions relating to the “*Minimum probability of transmission for endemization* ” scenario across 30 simulations. Furthermore, we have run new simulations of this scenario using four different values of *Juvenile-Threshold*, i.e., 500, 1000, 1500, and 2000 coexisting juvenile individuals, rather than just one value (1500), as in our previous version of the study (see also Reply 5 above). Results obtained using values ≥ 1000 confirmed the attainment of the endemic state with a probability of transmission per sexual contact of 70%, whereas a limit of 500 juvenile individuals only led to endemization of the STD in 74 out of 100 total simulations performed. Consequently, we can conclude that using small populations may have an impact on the system. We make clear this limitation in the new version of the manuscript (lines 431-433 and 687-702).

**Additional comments**

**Comment 11**: Would it not have been interesting to explore STDs that have a sterilising effect on males, i.e. wolbachia?

**Reply 11**: Although testing the effects of sterilising STDs on males was not the main focus of our study, we recognise the relevance of this question and believe that our model could easily simulate the fundamental epidemic dynamics of most systems in which both females and males may suffer a reduction in fertility caused by a pathogen. For instance, it has been reported that *Wolbachia* may also have a sterilising effect on females in *Aedes aegypti* [reference 1 in this document], which would make this particular case study suitable for investigation using the agent-based model presented in our manuscript. Indeed, as we pointed out in the Methods section (e.g., see lines 299-301), the health of male partners (in addition to that of females) is also an important variable affecting the final outcome of mating events in terms of offspring produced, even when the mother is healthy. We believe that the most appropriate way to interpret this reduction in our simulations is precisely as a decrease in male fecundity. This element of the model could simultaneously make some of our results more generalisable and serve as a basis for imagining alternative scenarios that focus more on the effects of STDs on males.

**Comment 12**: I don’t really understand the value add of using a spatial structure here. This is mentioned in the introduction as the value of this paper over network based models but it seems to include no features that couldn’t be simulated with a network model (albeit with difficulty). A network model would allow you to include more realistic distributions of mating (i.e. heavy tails). This could do with some exploration in the model, i.e. what is the actual distribution of mating events etc in your model.

**Reply 12**: We agree that, although potentially feasible, the spatial component of the model has not been explored extensively in the experimental scenarios presented in our study. It is also important to point out that there is a certain degree of overlap between many agent-based models and network-based models, in addition to the fact that an agent-based model can itself be based on a network. One of the characteristics of our model, which seems to be uncommon in this particular field, is that mating events are not implemented using networks (although this could potentially be done by increasing its level of sophistication). Although we concur that the spatial component is another facet of the model that could be explored further by performing some specifically designed scenarios or even by making it less abstract than it currently is, our intention in the Introduction (see lines 114-116) was mainly to highlight how even a model that is not based on networks can provide equally valid information on mating systems and STD epidemics, other than providing a platform to deepen these investigations from a spatial perspective.

**Comment 13**: This paper uses a lot of citations that don’t always directly address the claim being cited. These citations are often a decade or two old and are hard to access (even from a university with subscriptions to most journals). A single citation from a recent review or article (ideally from a open-access journal) would suffice and better represent the current state of the field.

**Reply 13**: We appreciate the reviewer’s concern regarding the citations. We agree that, where possible, citing recent and open-access reviews can help represent the current state of the field. However, we believe it is also important not to disregard older or less accessible references when they remain directly relevant to the questions addressed in our study. Some of these works continue to provide the foundation for current research. We have, nevertheless, revised the reference list and removed older citations that were not essential (specifically, we have removed 8 citations of works published before 1995). At the same time, we have added more recent references, including reviews where available, to provide a balanced and up-to-date representation of the literature.

**Comment 14**: Why isn’t the cost parameter and the benefit parameter a single parameter? Can’t this just be impact of mating on offspring count, it seems needlessly confusing?

**Reply 14**: The current version with separated costs and benefits of mating makes clearer that, although the relevant value for the simulations is of course the difference between these two parameters, the latter are still independent from each other for conceptual reasons. As a matter of fact, benefits and costs can be traced back to a wide variety of specific biological aspects that should be kept separate for greater clarity. This could make it easier to distinguish between all the factors involved, especially when it comes to explaining the initial conditions used and discussing the model’s outcomes.

**Comment 15**: Wouldn’t it be easier to express the time-frame in terms of generations or lifespan rather than constantly writing days/months/years?

**Reply 15**: We decided to use a continuous time-frame mainly because the structure of the model made this option the most appropriate. More specifically, from the early stages of implementation, it was clear that our simulations would always contain overlapping generations, in the first place because polyandrous females were programmed to produce their offspring in several batches separated in time, and the time between the production of one batch the next had to include a refractory period and the search for a new partner. This, especially considering females with a high degree of polyandry, made the non-overlapping generations option impractical, as well as the use of a discrete time-frame.

**Comment 16**: I found it quite hard to follow the interpretation of your results. It might be easier to read if you introduced the results and then discussed them before moving on the next set of simulations? Just a suggestion if that’s not against PLOS formatting rules.

**Reply 16**: We acknowledge that the results might be difficult to follow in some parts, mainly because of the number of experimental scenarios we presented in our manuscript. Consequently, in line with the author guidelines of PLoS ONE, we have decided to follow this suggestion and reorganise our manuscript by replacing the previous Results and Discussion sections with a single section entitled *Results and Discussion*.

**References cited in this document**

1. Osorio, J., Villa-Arias, S., Camargo, C. et al. wMel Wolbachia alters female post-mating behaviors and physiology in the dengue vector mosquito Aedes aegypti. Commun Biol 6, 865 (2023). https://doi.org/10.1038/s42003-023-05180-8
